# BNT162b2 Versus mRNA‐1273 Vaccines: Comparative Analysis of Long‐Term Protection Against SARS‐CoV‐2 Infection and Severe COVID‐19 in Qatar

**DOI:** 10.1111/irv.13357

**Published:** 2024-09-29

**Authors:** Hiam Chemaitelly, Houssein H. Ayoub, Peter Coyle, Patrick Tang, Mohammad R. Hasan, Hadi M. Yassine, Asmaa A. Al Thani, Zaina Al‐Kanaani, Einas Al‐Kuwari, Andrew Jeremijenko, Anvar Hassan Kaleeckal, Ali Nizar Latif, Riyazuddin Mohammad Shaik, Hanan F. Abdul‐Rahim, Gheyath K. Nasrallah, Mohamed Ghaith Al‐Kuwari, Adeel A. Butt, Hamad Eid Al‐Romaihi, Mohamed H. Al‐Thani, Abdullatif Al‐Khal, Roberto Bertollini, Laith J. Abu‐Raddad

**Affiliations:** ^1^ Infectious Disease Epidemiology Group, Weill Cornell Medicine–Qatar Cornell University Doha Qatar; ^2^ World Health Organization Collaborating Centre for Disease Epidemiology Analytics on HIV/AIDS, Sexually Transmitted Infections, and Viral Hepatitis, Weill Cornell Medicine–Qatar Cornell University, Qatar Foundation – Education City Doha Qatar; ^3^ Department of Population Health Sciences, Weill Cornell Medicine Cornell University New York New York USA; ^4^ Mathematics Program, Department of Mathematics and Statistics, College of Arts and Sciences Qatar University Doha Qatar; ^5^ Department of Biomedical Science, College of Health Sciences, QU Health Qatar University Doha Qatar; ^6^ Hamad Medical Corporation Doha Qatar; ^7^ Wellcome‐Wolfson Institute for Experimental Medicine Queens University Belfast UK; ^8^ Department of Pathology Sidra Medicine Doha Qatar; ^9^ Department of Pathology and Molecular Medicine McMaster University Hamilton Canada; ^10^ Biomedical Research Center, QU Health Qatar University Doha Qatar; ^11^ Department of Public Health, College of Health Sciences, QU Health Qatar University Doha Qatar; ^12^ Primary Health Care Corporation Doha Qatar; ^13^ Department of Medicine, Weill Cornell Medicine Cornell University New York New York USA; ^14^ Ministry of Public Health Doha Qatar; ^15^ College of Health and Life Sciences Hamad bin Khalifa University Doha Qatar

**Keywords:** cohort study, COVID‐19, epidemiology, immune imprinting, immunity, vaccine

## Abstract

**Background:**

This study provides a head‐to‐head comparison of the protection provided by the BNT162b2 and mRNA‐1273 vaccines against SARS‐CoV‐2 infection and against severe COVID‐19, covering primary series and third dose/booster vaccinations over up to 3 years of follow‐up, both before and after the emergence of the omicron variant.

**Methods:**

Two national, matched, retrospective cohort studies were conducted on Qatar's vaccinated population from December 16, 2020, to February 18, 2024. Subgroup analyses by pre‐vaccination SARS‐CoV‐2 infection history, as well as sensitivity analyses, were also conducted.

**Results:**

The adjusted hazard ratio (AHR) comparing infection incidence in those vaccinated with BNT162b2 versus mRNA‐1273 was 1.03 (95% CI: 1.02–1.05) after the primary series and 1.11 (95% CI: 1.09–1.13) after the third (booster) dose. The corresponding AHRs for any severe, critical, or fatal COVID‐19 were 1.31 (95% CI: 0.81–2.11) and 1.00 (95% CI: 0.20–4.94), respectively. Subgroup analyses by prior infection status hinted at a dose‐dependent immune imprinting effect, where a combination of two types of immunity, pre‐omicron and omicron, offered greater protection against infection than one type alone, with this effect being amplified by the higher antigen dose of mRNA‐1273 compared to BNT162b2. Sensitivity analyses confirmed the study findings.

**Conclusions:**

BNT162b2 provided slightly less protection against infection than mRNA‐1273 following both primary series and booster vaccinations while offering comparable protection against severe COVID‐19 outcomes. The findings suggested that the vaccine antigen dose in interaction with infection history may determine the extent of immune protection against infection.

## 1 Introduction

The introduction of mRNA vaccines, specifically BNT162b2 [1] (Pfizer‐BioNTech) and mRNA‐1273 [2] (Moderna), has played a critical role in curbing the spread of severe acute respiratory syndrome coronavirus 2 (SARS‐CoV‐2) and in reducing the morbidity and mortality associated with coronavirus disease 2019 (COVID‐19) [3, 4, 5, 6]. These vaccines initially demonstrated high efficacy in preventing SARS‐CoV‐2 infection and severe COVID‐19 in randomized clinical trials [1, 2] and in real‐world observational studies [3, 4, 5, 6]. However, the waning of their effectiveness over time [7, 8, 9, 10, 11], the emergence of new viral variants [11, 12, 13, 14, 15, 16], and the diverse history of natural infections in the population [12, 13, 14, 15, 16] can affect the vaccination outcomes and complicate our understanding of the vaccines' long‐term protection.

In the first study to provide a direct head‐to‐head comparison of the protection offered by two COVID‐19 vaccines, we evaluated the protection conferred by the primary series (two doses) of BNT162b2 versus the primary series of mRNA‐1273 during the initial 6 months post‐vaccination, at a time when the incidence was due to only pre‐omicron variants [17]. mRNA‐1273 was associated with a 30% lower incidence of SARS‐CoV‐2 infection compared to BNT162b2 [17], which aligns with the larger dose of the mRNA‐1273 vaccine [17] and the variations observed in neutralizing antibody titers [18]. Despite these differences, both vaccines provided strong protection against severe COVID‐19 outcomes, with no statistically significant difference between them [17].

Immune imprinting, a phenomenon where the specific sequence of immunological events (due to infection and/or vaccination) can enhance or compromise a person's future immune protection [19, 20, 21, 22, 23], might influence the effectiveness of vaccination. A series of laboratory and epidemiological studies have suggested that immune imprinting could affect the protection offered by vaccination and natural infection [19, 20, 21, 22, 23, 24, 25]. Notably, studies have indicated that a combination of pre‐omicron and omicron immunity, whether from vaccination or natural infection, provides greater protection against omicron infection than omicron immunity alone [20, 21]. This observation aligns with the notion that exposure to both pre‐omicron and omicron antigens broadens and strengthens the immune response against future infection challenges [20, 21]. Moreover, the imprinting effect could be more pronounced in individuals vaccinated with mRNA‐1273 than in those vaccinated with BNT162b2, possibly indicating a dose–response relationship for the imprinting effect [21].

In this study, we extended the follow‐up of our national cohorts in Qatar who received the BNT162b2 and mRNA‐1273 vaccines. This extended study (3 years of follow‐up) is 2.5 years longer than our first head‐to‐head comparison (6 months of follow‐up) [17] and captures the incidence of both pre‐omicron and omicron variants. Our goal is to assess long‐term differences in how these vaccines protect against SARS‐CoV‐2 infection and severe COVID‐19. We also compared the long‐term effects of a three‐dose regimen (primary series, followed by a booster) for these vaccines and explored potential immune imprinting by analyzing subgroups based on pre‐vaccination natural infection history (pre‐omicron and/or omicron).

## 2 Methods

### 2.1 Study Population, Data Sources, and Vaccination

This study was carried out among the resident population of Qatar from December 16, 2020, marking the start of the COVID‐19 vaccination campaign, to February 18, 2024, the study's end date. Data on COVID‐19 laboratory testing, vaccination, hospitalization, and death were retrieved from the integrated, nationwide digital health information platform (Appendix S1). These national federated databases contain all SARS‐CoV‐2‐related records, including vaccinations, hospitalizations, polymerase chain reaction (PCR) tests, irrespective of the location or facility, and, from January 5, 2022, all medically supervised rapid antigen tests (Appendix S2), with no missing information since the pandemic's onset. Until October 31, 2022, Qatar pursued an extensive testing strategy, testing 5% of the population weekly, mostly for routine purposes such as screening or travel‐related requirements [7, 13]. From November 1, 2022, onwards, testing was reduced to below 1% of the population weekly [26]. Most infections in Qatar were identified through routine testing rather than symptomatic presentation (Appendix S1) [7, 13].

Qatar initiated mass COVID‐19 vaccination on December 16, 2020, using BNT162b2 [5] and introduced mRNA‐1273 3 months later (Appendix S1) [6]. Vaccination was provided free of charge to all individuals, regardless of citizenship, exclusively through the public healthcare system [17]. Rollout prioritized frontline healthcare workers, individuals with severe or multiple chronic conditions, and individuals aged 50 years or older [7]. Vaccinations throughout the pandemic were administered adhering to the US Food and Drug Administration–approved protocol [1, 2].

Demographic information were obtained from the national health registry. Qatar's demographic composition is distinct with only 9% of the population aged 50 years or older and 89% being resident expatriates from over 150 countries [27]. Further details on Qatar's population and COVID‐19 databases have been previously published [4, 7, 13, 24, 27, 28, 29].

### 2.2 Study Design

This study conducted a head‐to‐head comparison of the incidence of infection and severe forms of COVID‐19 following primary series vaccination and a third (booster) dose vaccination with BNT162b2 compared to mRNA‐1273 using a matched retrospective cohort study design that emulates a randomized controlled trial (target trial design) [30, 31]. The first part of the study compared the incidence of infection in the national cohort of individuals who received the primary series vaccination with BNT162b2 to that in the national cohort of individuals who received the primary series vaccination with mRNA‐1273 (two‐dose analysis). The second part of the study replicated this analysis for the national cohorts of individuals who received three doses of each of these vaccines (three‐dose analysis).

Incidence of infection was defined as any PCR‐positive or rapid antigen–positive test after the start of follow‐up, regardless of symptoms. Infection severity classification followed the World Health Organization (WHO) guidelines for COVID‐19 case severity (acute care hospitalizations) [32], criticality (intensive care unit hospitalizations) [32], and fatality [33] (Appendix S3). Assessments were performed by trained medical personnel independent of study investigators using individual chart reviews.

As part of the national protocol, each individual with a SARS‐CoV‐2‐positive test and concurrent COVID‐19 hospital admission underwent an infection severity assessment every 3 days until discharge or death, irrespective of hospital length of stay. Individuals whose infection progressed to severe, critical, or fatal COVID‐19 were classified based on their worst outcome, starting with COVID‐19 death [33], followed by critical disease [32] and then severe disease [32] (Appendix S3). Incidence of severe COVID‐19 outcomes was recorded on the date of the SARS‐CoV‐2‐positive test confirming the infection.

### 2.3 Cohorts' Eligibility and Matching

Individuals were eligible for inclusion in the study if they had received two doses of either BNT162b2 or mRNA‐1273 for the two‐dose analysis and three doses of either vaccine for the three‐dose analysis. Individuals who received other vaccine types or mixed vaccines were excluded.

Individuals vaccinated with BNT162b2 were matched to individuals vaccinated with mRNA‐1273 exactly one to one by sex, 10‐year age group, nationality, number of coexisting conditions (ranging from 0 to ≥ 6; Appendix S4), prior infection status (no prior infection, prior pre‐omicron infection, prior omicron infection, or prior pre‐omicron and omicron infections), and calendar week of the second dose for the two‐dose analysis and calendar week of the third dose for the three‐dose analysis.

An iterative selection algorithm was implemented to ensure that, at the start of the follow‐up, matched pairs were alive, had the same vaccination status (primary series or three doses), had the same prior infection status, and had no documented SARS‐CoV‐2 infection within the previous 90 days (Appendix S5). The 90‐day threshold was used to avoid misclassification of a previous SARS‐CoV‐2 infection as an incident infection [13, 34, 35, 36]. Accordingly, a prior infection was defined as a SARS‐CoV‐2‐positive test ≥ 90 days before the start of follow‐up. Prior infections were classified as pre‐omicron whenever they occurred before December 19, 2021, the onset of the omicron wave in Qatar [13], and as omicron otherwise.

The matching strategy aimed to balance observed confounders that could potentially influence the risk of infection across the exposure groups [27, 37, 38, 39, 40]. The matching factors were selected based on findings from earlier COVID‐19 studies on Qatar's population [6, 7, 8, 16, 17, 41].

### 2.4 Cohorts' Follow‐Up

Follow‐up began 14 days after the second dose for the two‐dose analysis and 7 days after the third dose for the three‐dose analysis to allow for the buildup of immunity following vaccination. To ensure exchangeability [4, 42], both members of each matched pair were censored at the earliest occurrence of receiving an additional vaccine dose. Therefore, individuals were followed until the first of any of the following events: a documented SARS‐CoV‐2 infection (regardless of symptoms), third dose vaccination for those in the two‐dose analysis (with matched‐pair censoring), fourth dose vaccination for those in the three‐dose analysis (with matched‐pair censoring), death, or administrative end of follow‐up at end of the study.

### 2.5 Oversight

The institutional review boards at Hamad Medical Corporation and Weill Cornell Medicine–Qatar approved this retrospective study with a waiver of informed consent. The study was reported according to the Strengthening the Reporting of Observational Studies in Epidemiology (STROBE; Table S1).

### 2.6 Statistical Analysis

Eligible and matched cohorts were described using frequency distributions and measures of central tendency and were compared using standardized mean differences (SMDs). An SMD of ≤ 0.1 indicated adequate matching [43]. Cumulative incidence of infection (or severe, critical, or fatal COVID‐19), defined as the proportion of individuals at risk whose primary endpoint during follow‐up was an infection (or a severe COVID‐19 outcome), was estimated using the Kaplan–Meier estimator method. Schoenfeld residuals and log–log plots for survival curves were used to examine the proportional hazards assumption.

Incidence rate of infection (or severe COVID‐19 outcome) in each cohort, defined as number of identified infections (or severe COVID‐19 outcomes) divided by number of person‐weeks contributed by all individuals in the cohort, was estimated, with the corresponding 95% confidence interval (CI), using a Poisson log‐likelihood regression model with Stata 18.0 *stptime* command.

Overall adjusted hazard ratios (AHRs), comparing the incidence of infection (or severe COVID‐19 outcome) in the cohorts, and corresponding 95% CIs were calculated using Cox regression models with adjustment for the matching factors using the Stata 18.0 *stcox* command. The adjustment for the matching factors was implemented to ensure precise and unbiased estimation of standard variance [44]. These overall AHRs provided a weighted average of the time‐varying hazard ratios [45]. AHRs of infection were also estimated by 3‐month intervals from the start of follow‐up using separate Cox regressions, with “failure” restricted to specific time intervals. AHRs stratified by prior infection status were further calculated. CIs were not adjusted for multiplicity.

A sensitivity analysis was conducted by further adjusting the AHRs for differences in testing rate between the study cohorts. Low testers were defined as people having < 3 tests per person‐year, intermediate testers having 3–4 tests per person‐year, and high testers having ≥ 5 tests per person‐year, by the end of study.

The study's methodology involved creating matched cohorts designed for straightforward disaggregation to facilitate subgroup analyses. This strategy carries the potential drawback of generating population compositions that may not accurately reflect the intended target groups. To assess whether this approach could have impacted the study's findings, we performed an additional sensitivity analysis. This involved rederiving the AHRs for the subgroup analyses by incorporating interaction terms between the study cohorts and prior infection statuses.

Statistical analyses were performed using Stata/SE Version 18.0 (Stata Corporation, College Station, TX, USA).

## 3 Results

### 3.1 Study Population

Between December 16, 2020, and February 18, 2024, 1,322,715 individuals in the total population of Qatar received at least two doses of BNT162b2, of whom 448,322 received a third dose. The median dates for the first, second, and third doses were May 3, 2021, May 25, 2021, and January 7, 2022, respectively. The median time between the first and second doses was 21 days (interquartile range [IQR], 21–22 days). The median time between the second and third doses was 256 days (IQR, 236–287 days).

Over the same duration, 907,269 individuals in the total population of Qatar received at least two doses of mRNA‐1273, of whom 241,601 received a third dose. The median dates for the first, second, and third doses were May 28, 2021, June 28, 2021, and February 13, 2022, respectively. The median time between the first and second doses was 28 days (IQR, 28–30 days). The median time between the second and third doses was 250 days (IQR, 219–287 days).

Figure S1 illustrates the study population selection process. Table 1 describes the characteristics of the full and matched cohorts. This study was conducted on Qatar's entire population, and therefore, the study population is representative of the country's internationally diverse, predominantly young and male, demographic profile.

**TABLE 1:** Baseline characteristics of the full and matched cohorts for the BNT162b2 and mRNA‐1273 vaccines in the two‐dose and three‐dose analyses.

	Two‐dose analysis						Three‐dose analysis					
Characteristics	Full eligible cohorts			Matched cohorts^a^			Full eligible cohorts			Matched cohorts^a^		
	BNT162b2	mRNA‐1273	SMD^b^	BNT162b2	mRNA‐1273	SMD^b^	BNT162b2	mRNA‐1273	SMD^b^	BNT162b2	mRNA‐1273	SMD^b^
	*N* = 1,287,695	*N* = 907,257		*N* = 390,495	N = 390,495		*N* = 430,475	*N* = 241,600		*N* = 177,422	N = 177,422	
Median age (IQR)—years	38 (30–46)	37 (32–44)	0.00^c^	37 (31–44)	37 (31–44)	0.01^c^	41 (33–51)	39 (33–46)	0.11^c^	39 (34–46)	39 (34–47)	0.00^c^
Age group—*n* (%)												
0–19 years	107,719 (8.4)	87 (0.01)	0.50	70 (0.02)	70 (0.02)	0.00	33,067 (7.7)	11 (< 0.01)	0.54	6 (< 0.01)	6 (< 0.01)	0.00
20–29 years	188,720 (14.7)	144,755 (16.0)		69,348 (17.8)	69,348 (17.8)		38,766 (9.0)	28,400 (11.8)		19,264 (10.9)	19,264 (10.9)	
30–39 years	422,793 (32.8)	399,324 (44.0)		160,461 (41.1)	160,461 (41.1)		125,108 (29.1)	97,428 (40.3)		69,896 (39.4)	69,896 (39.4)	
40–49 years	335,765 (26.1)	245,558 (27.1)		115,539 (29.6)	115,539 (29.6)		117,565 (27.3)	73,546 (30.4)		57,151 (32.2)	57,151 (32.2)	
50–59 years	152,357 (11.8)	93,468 (10.3)		36,840 (9.4)	36,840 (9.4)		70,757 (16.4)	32,489 (13.4)		24,514 (13.8)	24,514 (13.8)	
60–69 years	59,865 (4.6)	20,522 (2.3)		6970 (1.8)	6970 (1.8)		33,819 (7.9)	8500 (3.5)		5975 (3.4)	5975 (3.4)	
70+ years	20,476 (1.6)	3543 (0.4)		1267 (0.3)	1267 (0.3)		11,393 (2.6)	1226 (0.5)		616 (0.3)	616 (0.3)	
Sex												
Male	891,624 (69.2)	729,109 (80.4)	0.26	311,018 (79.6)	311,018 (79.6)	0.00	275,398 (64.0)	165,527 (68.5)	0.10	124,269 (70.0)	124,269 (70.0)	0.00
Female	396,071 (30.8)	178,148 (19.6)		79,477 (20.4)	79,477 (20.4)		155,077 (36.0)	76,073 (31.5)		53,153 (30.0)	53,153 (30.0)	
Nationality^d^												
Bangladeshi	139,776 (10.9)	169,640 (18.7)	0.57	66,616 (17.1)	66,616 (17.1)	0.00	30,137 (7.0)	29,249 (12.1)	0.47	19,595 (11.0)	19,595 (11.0)	0.00
Egyptian	71,897 (5.6)	35,724 (3.9)		15,366 (3.9)	15,366 (3.9)		36,387 (8.5)	18,211 (7.5)		13,827 (7.8)	13,827 (7.8)	
Filipino	119,203 (9.3)	85,168 (9.4)		37,146 (9.5)	37,146 (9.5)		56,564 (13.1)	36,357 (15.0)		27,479 (15.5)	27,479 (15.5)	
Indian	288,307 (22.4)	252,005 (27.8)		106,676 (27.3)	106,676 (27.3)		127,486 (29.6)	84,432 (34.9)		68,008 (38.3)	68,008 (38.3)	
Nepalese	110,508 (8.6)	125,933 (13.9)		55,161 (14.1)	55,161 (14.1)		14,195 (3.3)	12,564 (5.2)		9484 (5.3)	9484 (5.3)	
Pakistani	56,036 (4.4)	48,752 (5.4)		19,909 (5.1)	19,909 (5.1)		18,222 (4.2)	13,401 (5.5)		8514 (4.8)	8514 (4.8)	
Qatari	179,084 (13.9)	16,673 (1.8)		15,838 (4.1)	15,838 (4.1)		37,180 (8.6)	2330 (1.0)		2034 (1.1)	2034 (1.1)	
Sri Lankan	39,340 (3.1)	37,428 (4.1)		16,729 (4.3)	16,729 (4.3)		10,825 (2.5)	8726 (3.6)		6214 (3.5)	6214 (3.5)	
Sudanese	29,449 (2.3)	16,283 (1.8)		7780 (2.0)	7780 (2.0)		8551 (2.0)	3432 (1.4)		2297 (1.3)	2297 (1.3)	
Other nationalities^e^	254,095 (19.7)	119,651 (13.2)		49,274 (12.6)	49,274 (12.6)		90,928 (21.1)	32,898 (13.6)		19,970 (11.3)	19,970 (11.3)	
Coexisting conditions												
0	1,021,988 (79.4)	810,166 (89.3)	0.29	349,235 (89.4)	349,235 (89.4)	0.00	306,774 (71.3)	198,235 (82.1)	0.28	149,113 (84.0)	149,113 (84.0)	0.00
1	131,483 (10.2)	54,078 (6.0)		24,249 (6.2)	24,249 (6.2)		53,302 (12.4)	21,708 (9.0)		14,803 (8.3)	14,803 (8.3)	
2	63,045 (4.9)	24,782 (2.7)		10,338 (2.6)	10,338 (2.6)		30,630 (7.1)	12,070 (5.0)		8027 (4.5)	8027 (4.5)	
3	30,660 (2.4)	9771 (1.1)		3722 (1.0)	3722 (1.0)		16,751 (3.9)	5136 (2.1)		3202 (1.8)	3202 (1.8)	
4	18,434 (1.4)	4565 (0.5)		1602 (0.4)	1602 (0.4)		10,545 (2.4)	2527 (1.0)		1409 (0.8)	1409 (0.8)	
5	10,880 (0.8)	2242 (0.2)		707 (0.2)	707 (0.2)		6199 (1.4)	1196 (0.5)		548 (0.3)	548 (0.3)	
≥ 6	11,205 (0.9)	1653 (0.2)		642 (0.2)	642 (0.2)		6274 (1.5)	728 (0.3)		320 (0.2)	320 (0.2)	
Prior infection status^f^												
No prior infection	1,156,620 (89.8)	—	—	351,127 (89.9)	351,127 (89.9)	0.00	362,432 (84.2)	—	—	150,086 (84.6)	150,086 (84.6)	0.00
Prior pre‐omicron infection	129,951 (10.1)	—		39,154 (10.0)	39,154 (10.0)		58,351 (13.6)	—		23,021 (13.0)	23,021 (13.0)	
Prior omicron infection	1031 (0.1)	—		205 (0.1)	205 (0.1)		8751 (2.0)	—		4027 (2.3)	4027 (2.3)	
Prior pre‐omicron and omicron infections	93 (0.01)	—		9 (< 0.01)	9 (< 0.01)		941 (0.2)	—		288 (0.2)	288 (0.2)	

Abbreviations: IQR, interquartile range; SMD, standardized mean difference.

^a^ Cohorts were matched one to one by sex, 10‐year age group, nationality, prior infection status, and calendar week of the second vaccine dose in the two‐dose analysis and of the third vaccine dose in the three‐dose analysis.

^b^ SMD is the difference in the mean of a covariate between groups divided by the pooled standard deviation. An SMD < 0.1 indicates adequate matching.

^c^ SMD is for the mean difference between groups divided by the pooled standard deviation.

^d^ Nationalities were chosen to represent the most populous groups in Qatar.

^e^ These comprise up to 169 other nationalities in the unmatched and 123 other nationalities in the matched two‐dose analyses and up to 165 other nationalities in the unmatched and 107 other nationalities in the matched three‐dose analyses.

^f^ Ascertained at the start of follow‐up. Accordingly, distribution is not available for the unmatched mRNA‐1273 cohorts, as the start of follow‐up for each person in the mRNA‐1273 cohort is determined by that of their match in the BNT162b2 cohort after the matching process is completed.

### 3.2 Two‐Dose Analysis

**FIGURE 1:**
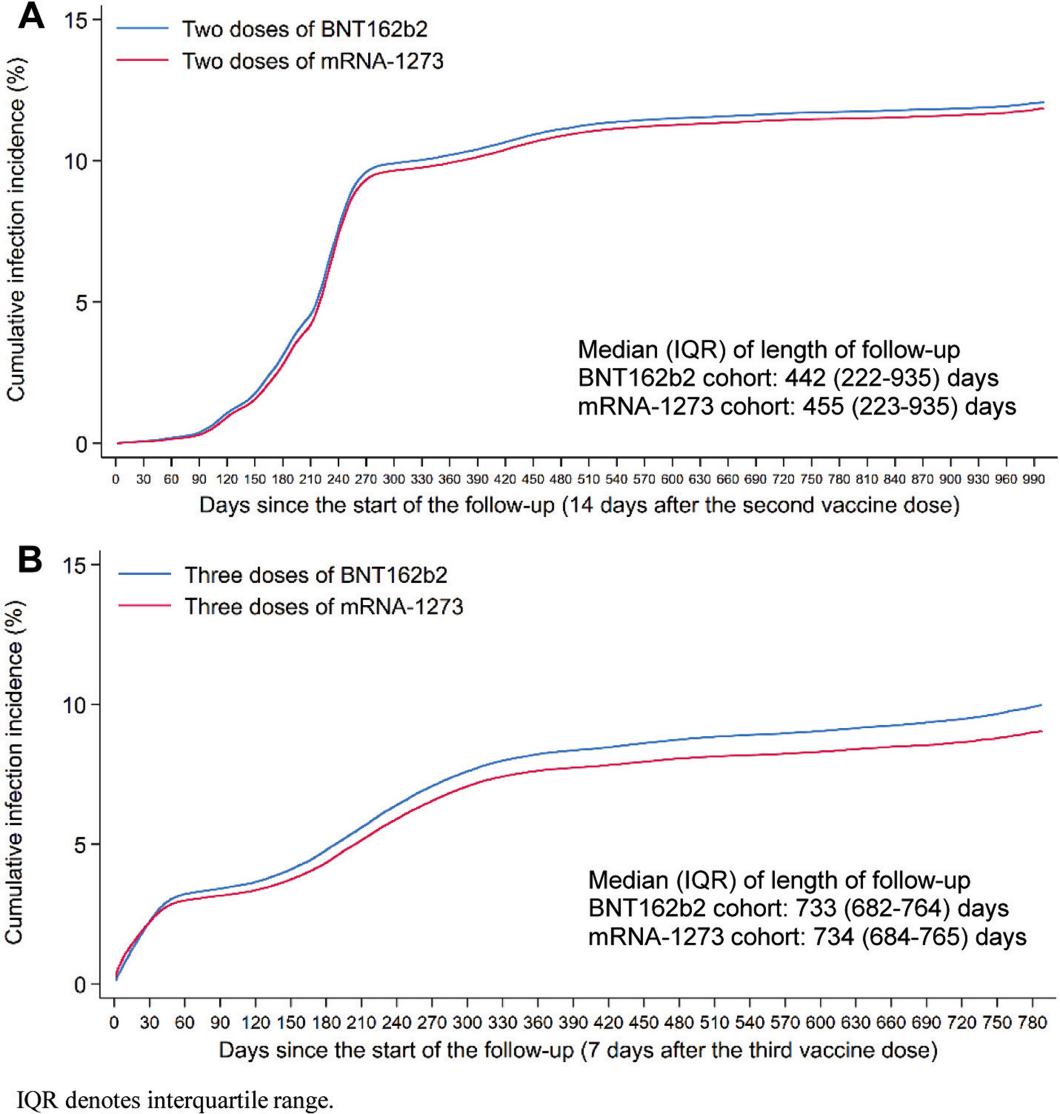
Cumulative incidence of SARS‐CoV‐2 infection after (A) two doses and (B) three doses of the BNT162b2 and mRNA‐1273 vaccines.

A total of 37,097 infections were recorded in the BNT162b2 matched study cohort at least 14 days after receiving the second dose (Figure S1 and Table 2A). Of these infections, 32 progressed to severe, 7 to critical, and 0 to fatal COVID‐19. Meanwhile, 36,076 infections were recorded in the mRNA‐1273 cohort, of which 20 progressed to severe, 8 to critical, and 2 to fatal COVID‐19. The median time of follow‐up was 442 days (IQR, 222–935 days) for the BNT162b2 cohort and 455 days (IQR, 223–935 days) for the mRNA‐1273 cohort (Figure 1A).

**TABLE 2:** Hazard ratios for incidence of SARS‐CoV‐2 infection and of severe, critical, or fatal COVID‐19 in the (A) two‐dose analysis and (B) three‐dose analysis.

A. Two‐dose analysis	BNT162b2 cohort^a^	mRNA‐1273 cohort^a^
Main analysis		
Sample size	390,495	390,495
Number of incident infections	37,097	36,076
Total follow‐up time (person‐weeks)	32,081,665	32,160,114
Incidence rate of infection (per 10,000 person‐weeks; 95% CI)	11.6 (11.5–11.7)	11.2 (11.1–11.3)
Unadjusted hazard ratio for SARS‐CoV‐2 infection (95% CI)	1.03 (1.02–1.05)	
Adjusted hazard ratio for SARS‐CoV‐2 infection (95% CI)^b^	1.03 (1.02–1.05)	
Unadjusted hazard ratio for severe, critical, or fatal COVID‐19 disease (95% CI)	1.30 (0.81–2.10)	
Adjusted hazard ratio for severe, critical, or fatal COVID‐19 disease (95% CI)^b^	1.31 (0.81–2.11)	
Subgroup analyses		
No prior infection		
Incidence rate of infection (per 10,000 person‐weeks; 95% CI)	11.7 (11.6–11.9)	11.4 (11.3–11.5)
Unadjusted hazard ratio for SARS‐CoV‐2 infection (95% CI)	1.03 (1.01–1.04)	
Adjusted hazard ratio for SARS‐CoV‐2 infection (95% CI)^b^	1.03 (1.02–1.05)	
Prior pre‐omicron infection		
Incidence rate of infection (per 10,000 person‐weeks; 95% CI)	9.9 (9.6–10.3)	9.5 (9.2–9.8)
Unadjusted hazard ratio for SARS‐CoV‐2 infection (95% CI)	1.05 (1.00–1.10)	
Adjusted hazard ratio for SARS‐CoV‐2 infection (95% CI)^b^	1.05 (1.00–1.10)	
Prior omicron infection		
Incidence rate of infection (per 10,000 person‐weeks; 95% CI)	7.6 (4.2–13.7)	3.4 (1.4–8.2)
Unadjusted hazard ratio for SARS‐CoV‐2 infection (95% CI)	2.23 (0.78–6.43)	
Adjusted hazard ratio for SARS‐CoV‐2 infection (95% CI)^b^	2.43 (0.84–7.02)	
Prior pre‐omicron and omicron infections		
Incidence rate of infection (per 10,000 person‐weeks; 95% CI)	—	—
Unadjusted hazard ratio for SARS‐CoV‐2 infection (95% CI)	—	
Adjusted hazard ratio for SARS‐CoV‐2 infection (95% CI)^b^	—	

B. Three‐dose analysis	BNT162b2 cohort^a^	mRNA‐1273 cohort^a^
Main analysis		
Sample size	177,422	177,422
Number of incident infections	16,907	15,405
Total follow‐up time (person‐weeks)	16,947,840	17,048,557
Incidence rate of infection (per 10,000 person‐weeks; 95% CI)	10.0 (9.8–10.1)	9.0 (8.9–9.2)
Unadjusted hazard ratio for SARS‐CoV‐2 infection (95% CI)	1.10 (1.08–1.13)	
Adjusted hazard ratio for SARS‐CoV‐2 infection (95% CI)^b^	1.11 (1.09–1.13)	
Unadjusted hazard ratio for severe, critical, or fatal COVID‐19 disease (95% CI)	1.00 (0.20–4.97)	
Adjusted hazard ratio for severe, critical, or fatal COVID‐19 disease (95% CI)	1.00 (0.20–4.94)	
Subgroup analyses		
No prior infection		
Incidence rate of infection (per 10,000 person‐weeks; 95% CI)	10.3 (10.1–10.4)	9.3 (9.1–9.4)
Unadjusted hazard ratio for SARS‐CoV‐2 infection (95% CI)	1.10 (1.08–1.13)	
Adjusted hazard ratio for SARS‐CoV‐2 infection (95% CI)^b^	1.11 (1.09–1.14)	
Prior pre‐omicron infection		
Incidence rate of infection (per 10,000 person‐weeks; 95% CI)	8.4 (8.0–8.7)	8.2 (7.8–8.6)
Unadjusted hazard ratio for SARS‐CoV‐2 infection (95% CI)	1.02 (0.96–1.09)	
Adjusted hazard ratio for SARS‐CoV‐2 infection (95% CI)^b^	1.02 (0.96–1.09)	
Prior omicron infection		
Incidence rate of infection (per 10,000 person‐weeks; 95% CI)	8.9 (8.0–10.0)	5.7 (4.9–6.5)
Unadjusted hazard ratio for SARS‐CoV‐2 infection (95% CI)	1.58 (1.32–1.89)	
Adjusted hazard ratio for SARS‐CoV‐2 infection (95% CI)^b^	1.59 (1.33–1.90)	
Prior pre‐omicron and omicron infections		
Incidence rate of infection (per 10,000 person‐weeks; 95% CI)	10.4 (7.1–15.3)	5.8 (3.5–9.7)
Unadjusted hazard ratio for SARS‐CoV‐2 infection (95% CI)	1.78 (0.94–3.36)	
Adjusted hazard ratio for SARS‐CoV‐2 infection (95% CI)^b^	1.97 (1.04–3.73)	

Abbreviations: CI, confidence interval; COVID‐19, coronavirus disease 2019; SARS‐CoV‐2, severe acute respiratory syndrome coronavirus 2.

^a^ Cohorts were matched exactly one to one by sex, 10‐year age group, nationality, number of coexisting conditions, prior infection status, and calendar week of the second vaccine dose in the two‐dose analysis and calendar week of the third dose in the three‐dose analysis.

^b^ Adjusted for sex, 10‐year age group, nationality, number of coexisting conditions, and calendar week of the second vaccine dose in the two‐dose analysis and calendar week of the third dose in the three‐dose analysis.

Cumulative incidence of infection was 12.0% (95% CI: 11.9–12.2%) for the BNT162b2 cohort and 11.8% (95% CI: 11.7–11.9%) for the mRNA‐1273 cohort, 990 days after the start of follow‐up (Figure 1A). The overall AHR comparing incidence of infection in the BNT162b2 cohort to the mRNA‐1273 cohort—controlling for sex, 10‐year age group, nationality group, number of coexisting conditions, prior infection status, and calendar week of the second vaccine dose—was estimated at 1.03 (95% CI: 1.02–1.05; Table 2A). The overall AHR for any severe, critical, or fatal COVID‐19 was estimated at 1.31 (95% CI: 0.81–2.11).

**FIGURE 2:**
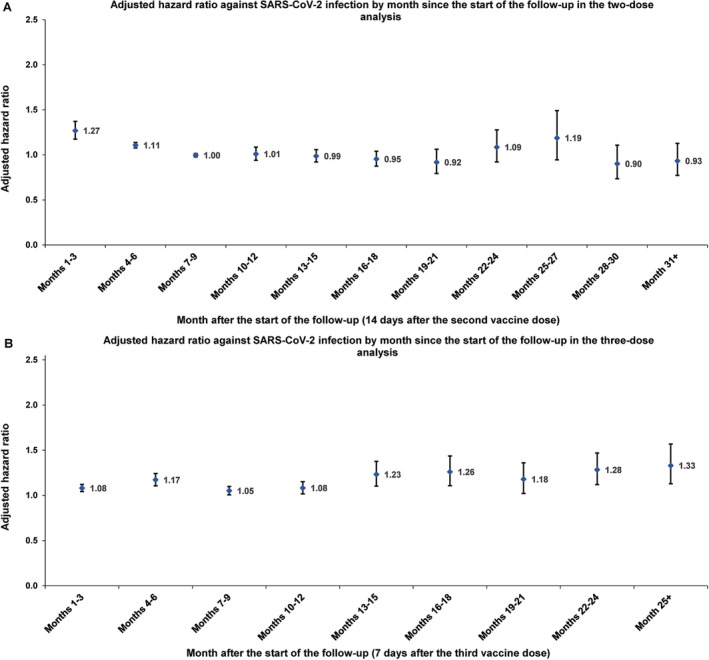
Adjusted hazard ratios for incidence of SARS‐CoV‐2 infection in the matched (A) primary series BNT162b2 cohort versus primary series mRNA‐1273 cohort and (B) three‐dose BNT162b2 cohort versus three‐dose mRNA‐1273 cohort, by month of follow‐up.

The AHR by time since the second dose was highest within the first 3 months (Figure 2A). At this peak, BNT162b2 offered about 30% less protection compared to mRNA‐1273, as indicated by the AHR of 1.27 (95% CI: 1.17–1.37). This initial difference narrowed in the following 3 months, with the AHR dropping to 1.11 (95% CI: 1.08–1.14). Thereafter, the AHR remained close to 1 throughout the follow‐up period, although there was a slight, temporary increase in the AHR around the 2‐year mark of follow‐up. This modest increase was observed primarily during the spring and summer of 2023, a period throughout which the incidence was predominantly driven by XBB subvariants (Figure S2).

The subgroup analysis by prior infection status estimated the AHR at 1.03 (95% CI: 1.02–1.05) for individuals with no prior infection, 1.05 (95% CI: 1.00–1.10) for those with a prior pre‐omicron infection, and 2.43 (95% CI: 0.84–7.02) for those with a prior omicron infection (Table 2A). It is of note that the latter estimate was based on a small cohort of 205 individuals in each arm, as the vast majority of individuals received their second vaccine dose before the onset of the omicron wave in Qatar.

The sensitivity analysis additionally adjusting estimates for differences in testing rate between the study cohorts yielded an overall AHR of 1.03 (95% CI: 1.01–1.04) and subgroup AHRs of 1.03 (95% CI: 1.01–1.04) for individuals with no prior infection, 1.03 (95% CI: 0.98–1.08) for those with a pre‐omicron infection, and 2.43 (95% CI: 0.84–7.02) for those with a prior omicron infection (Table S2A).

The sensitivity analysis using interaction terms between study cohort and prior infection status (instead of cohort disaggregation in the main analysis) yielded AHR estimates of 1.03 (95% CI: 1.02–1.05) for individuals with no prior infection, 1.05 (95% CI: 1.00–1.10) for those with a pre‐omicron infection, and 2.26 (95% CI: 0.78–6.49) for those with a prior omicron infection (Table S2A).

### 3.3 Three‐Dose Analysis

A total of 16,907 infections were recorded in the BNT162b2 matched study cohort at least 7 days after receiving the third dose (Figure S1 and Table 2B). Of these infections, three progressed to severe, but none to critical or fatal COVID‐19. Meanwhile, 15,405 infections were recorded in the mRNA‐1273 cohort, of which one progressed to severe, one to critical, and one to fatal COVID‐19. The median time of follow‐up was 733 days (IQR, 682–764 days) for the BNT162b2 cohort and 734 days (IQR, 684–765 days) for the mRNA‐1273 cohort (Figure 1B).

Cumulative incidence of infection was 9.9% (95% CI: 9.8–10.1%) for the BNT162b2 cohort and 9.0% (95% CI: 8.9–9.1%) for the mRNA‐1273 cohort, 780 days after the start of follow‐up (Figure 1B). The overall AHR comparing incidence of infection in the BNT162b2 cohort to the mRNA‐1273 cohort—controlling for sex, 10‐year age group, nationality group, number of coexisting conditions, prior infection status, and calendar week of the third vaccine dose—was estimated at 1.11 (95% CI: 1.09–1.13; Table 2B). The overall AHR for any severe, critical, or fatal COVID‐19 was estimated at 1.00 (95% CI: 0.20–4.94). The AHR by time since the third dose remained above 1 throughout the follow‐up period (Figure 2B). It also seemed to increase slightly over time, reaching an AHR of approximately 1.3.

The subgroup analysis by prior infection status estimated the AHR at 1.11 (95% CI: 1.09–1.14) for individuals with no prior infection, 1.02 (95% CI: 0.96–1.09) for those with a prior pre‐omicron infection, 1.59 (95% CI: 1.33–1.90) for those with a prior omicron infection, and 1.97 (95% CI: 1.04–3.73) for those with prior pre‐omicron and omicron infections (Table 2B).

The sensitivity analysis additionally adjusting estimates for differences in testing rate between the study cohorts yielded an overall AHR of 1.05 (95% CI: 1.03–1.07) and subgroup AHRs of 1.05 (95% CI: 1.03–1.08) for individuals with no prior infection, 0.98 (95% CI: 0.93–1.05) for those with a pre‐omicron infection, 1.40 (95% CI: 1.17–1.68) for those with a prior omicron infection, and 1.92 (95% CI: 1.00–3.69) for those with prior pre‐omicron and omicron infections (Table S2B).

The sensitivity analysis using interaction terms between study cohort and prior infection status (instead of cohort disaggregation in the main analysis) yielded AHR estimates of 1.11 (95% CI: 1.09–1.14) for individuals with no prior infection, 1.02 (95% CI: 0.96–1.09) for those with a pre‐omicron infection, 1.58 (95% CI: 1.32–1.88) for those with a prior omicron infection, and 1.77 (95% CI: 0.94–3.35) for those with prior pre‐omicron and omicron infections (Table S2B).

## 4 Discussion

The results indicated that individuals vaccinated with mRNA‐1273 experienced a lower incidence of infection compared to those vaccinated with BNT162b2, both after the primary series and following the third/booster dose. Notably, for the primary series, most of this difference was observed in the initial months following the second dose before the waning of antibodies [46, 47]. This observation is consistent with our previous analysis, which was restricted to the early post‐vaccination period [17]. The difference in incidence rates between the vaccines may be attributed to the higher antigen dose in the mRNA‐1273 vaccine (100 μg) [2] compared to the BNT162b2 vaccine (30 μg) [1], which appears to result in variations in neutralizing antibody titers [18].

For the third dose, the observed difference in protection against infection was smaller immediately after dose administration, which could be attributed to the mRNA‐1273 booster dose being half that of the primary series (50 μg vs. 100 μg) [2, 4] and the BNT162b2 booster dose remaining equal to that of the primary series (30 μg) [1, 4]. However, this difference in protection against infection not only persisted but also appeared to increase over the follow‐up period post‐booster. Despite these (generally modest) differences in protection against infection between these two vaccines, there was no evidence of differences in their effectiveness against severe, critical, or fatal COVID‐19 outcomes either after the primary series or after the booster dose.

The results indirectly hinted at a dose‐dependent immune imprinting effect: Combined pre‐omicron and omicron immunity (achieved through vaccination or natural infection) offers greater protection against infection than pre‐omicron immunity alone [20, 21], with this effect amplified by a higher antigen dose (that of mRNA‐1273 [2]). For those with a prior pre‐omicron infection before vaccination, the aHR was 1.05 (95% CI: 1.00–1.10) in the two‐dose analysis and 1.02 (95% CI: 0.96–1.09) in the three‐dose analysis (Table 2). Meanwhile, for those with a prior omicron infection before vaccination, the aHRs were substantially larger at 2.43 (95% CI: 0.84–7.02) in the two‐dose analysis and 1.59 (95% CI: 1.33–1.90) in the three‐dose analysis. This effect, observed in both the two‐dose and three‐dose cohorts, did not reach statistical significance in the two‐dose analysis but is consistent with observations from our earlier studies of immune imprinting [20, 21]. This suggests a dose‐dependent effect, as the difference between these two vaccines is related to antigen dose, with both otherwise having a similar design [1, 2]. The two sensitivity analyses corroborated these findings; however, further studies are warranted to definitively confirm and elucidate this dose‐dependent effect.

The slowly increasing difference in protection observed in the three‐dose analysis (Figure 2B), as well as the apparent increase during later follow‐up periods in the two‐dose analysis (Figure 2A), may also be attributed to this immune imprinting effect. This hypothesis is supported by the fact that these later follow‐up periods coincided with times of reduced testing. Consequently, it is possible that many cohort members experienced increasing numbers of undocumented omicron infections post‐vaccination, which could result in a progressively larger observed difference in protection.

The study findings may have implications for other infections and future pandemics. First, vaccine antigen dose appears to play a role in determining vaccine effectiveness against infection [48], but it is also important to consider whether a higher antigen dose may increase the risk of adverse events [49]. Second, although protection against infection varied across vaccines, the differences in protection against severe forms of COVID‐19 were not clinically large and were not statistically significant. This suggests that future pandemic strategies should focus on maximizing vaccine coverage rather than prioritizing specific vaccines to optimize rapid protection against severe disease in the population. Third, the study highlighted the utility of the target trial design in evaluating differences in the effectiveness of vaccines and their long‐term impacts in real‐world settings. This approach, suitable for both emerging and endemic infections, allows for rapid assessments and may reduce the need for time‐consuming and resource‐intensive randomized controlled trials [30, 31].

This study has limitations. This study relied on documented SARS‐CoV‐2 infections to compare incidence between cohorts. However, many infections, particularly since the testing reduction on November 1, 2022, might be undocumented. Although documented SARS‐CoV‐2 infections were considered a proxy for incidence, this measure actually reflects both incidence (symptom‐driven testing) and prevalence (routine/random testing) of infection. However, the study outcomes are relative measures comparing the two cohorts and therefore should not be impacted by under‐ascertainment of incident infections as long as under‐ascertainment does not differentially affect the compared cohorts.

Testing frequency may differ between cohorts, suggesting potential differential outcome ascertainment. Nevertheless, the sensitivity analysis, which adjusted estimates for differences in testing rates between cohorts, affirmed the main study findings. The study also matched observable confounders across cohorts to control for any potential effects of differences in testing across confounder values. Home‐based rapid antigen testing is not documented (Appendix S1) and is not factored in these analyses. However, there is no reason to believe that home‐based testing could have differentially affected the followed cohorts to alter study estimates. Matching was done while factoring key socio‐demographic characteristics of the population, and this may also have controlled or reduced differences in home‐based testing between cohorts.

With the relatively young population of Qatar [27], our findings may not be generalizable to other countries where elderly citizens constitute a large proportion of the population. The all‐cause mortality database used in the analyses was complete up to June 15, 2023, but it missed non‐COVID‐19 deaths after this date. However, this is unlikely to have impacted the results, considering the very low mortality rate in this young and healthy population [28, 29]. Our previous cohort studies have consistently demonstrated limited censoring due to deaths [4, 24, 28, 29, 50].

Qatar has diverse demographics, with 89% of the population being expatriates from over 150 countries [27]. Data on the travel history of the study population were not available. Given the high proportion of expatriates, it is plausible that the rate of travel is higher than in other countries. To account for this, matching by nationality, age, and sex was performed to balance travel rates across the cohorts. These demographic factors serve as strong proxies for socioeconomic status and occupation in this country [27, 38, 40] and consequently potentially for the rate of travel outside the country. Although matching can impact the representativeness of the study population, differences between the matched and fully eligible cohorts were minor in this study (Table 1). The matched cohorts were broadly representative of Qatar's population structure, which primarily consists of young expatriates [27, 38, 40].

Although robust matching was implemented, the availability of data limited matching on other factors such as geography or occupation. However, being essentially a city‐state, infection incidence in Qatar was broadly distributed across neighborhoods. Nationality, age, and sex serve as powerful proxies for socioeconomic status in this country [27, 37, 38, 39, 40], and thus, matching by these factors may have also, at least partially, controlled for other variables such as occupation. This matching approach has been previously investigated in studies with different epidemiologic designs using control groups to test for null effects [6, 7, 8, 17, 51]. This includes our earlier comparison of these two vaccines, which showed no difference in infection incidence during the first 2 weeks after the first dose [17]. These studies have supported the effectiveness of this matching prescription in controlling for differences in infection exposure [6, 7, 8, 17, 51]. However, bias in real‐world data can arise unexpectedly or from unknown sources, such as subtle behavioral differences, variations in test accessibility, or policy shifts related to testing or vaccination privileges, among other factors.

Due to the low number of severe, critical, or fatal infections among these vaccinated cohorts, we were unable to conduct separate analyses for each severity category (severe, critical, or fatal COVID‐19). Although there were variations in the distribution of these outcomes across cohorts, the limited number of cases suggests that these differences likely arose by chance.

The study has strengths. It was conducted on a large national scale, encompassing a diverse population based on national backgrounds and utilized extensive, validated databases established through numerous COVID‐19 studies. The follow‐up period was long, spanning several years after vaccination. Exact matching was employed to ensure rigorous pairing of cohorts. Finally, estimates were confirmed through sensitivity analyses, which adjusted for differences in testing rates between the cohorts and employed alternative analysis methodologies for estimating study outcomes, namely, the use of interaction terms.

In conclusion, BNT162b2 showed less protection against infection compared to mRNA‐1273 following both the primary series and booster vaccinations, perhaps reflecting the differences in antigen doses of the two vaccines. However, no significant differences were observed in effectiveness against severe outcomes following both the primary series and booster vaccinations. The results hinted at a dose‐dependent immune imprinting effect, where a combination of two types of immunity, pre‐omicron and omicron, offered greater protection against infection than one type alone, with this effect being amplified by the higher antigen dose of mRNA‐1273 compared to BNT162b2.

## Author Contributions

**Hiam Chemaitelly:** conceptualization, data curation, formal analysis, funding acquisition, investigation, methodology, project administration, resources, software, supervision, validation, writing – original draft, writing – review and editing. **Houssein H. Ayoub:** data curation, funding acquisition, resources, writing – review and editing. **Peter Coyle:** data curation, funding acquisition, resources, writing – review and editing. **Patrick Tang:** data curation, funding acquisition, resources, writing – review and editing. **Mohammad R. Hasan:** data curation, funding acquisition, resources, writing – review and editing. **Hadi M. Yassine:** data curation, funding acquisition, writing – review and editing, resources. **Asmaa A. Al Thani:** data curation, funding acquisition, writing – review and editing, resources. **Zaina Al‐Kanaani:** data curation, funding acquisition, resources, writing – review and editing. **Einas Al‐Kuwari:** data curation, resources, writing – review and editing, funding acquisition. **Andrew Jeremijenko:** data curation, resources, writing – review and editing, funding acquisition. **Anvar Hassan Kaleeckal:** data curation, resources, writing – review and editing, funding acquisition. **Ali Nizar Latif:** funding acquisition, writing – review and editing, data curation, resources. **Riyazuddin Mohammad Shaik:** data curation, resources, writing – review and editing, funding acquisition. **Hanan F. Abdul‐Rahim:** data curation, resources, funding acquisition, writing – review and editing. **Gheyath K. Nasrallah:** data curation, resources, writing – review and editing, funding acquisition. **Mohamed Ghaith Al‐Kuwari:** data curation, resources, funding acquisition, writing – review and editing. **Adeel A. Butt:** data curation, resources, writing – review and editing, funding acquisition. **Hamad Eid Al‐Romaihi:** data curation, resources, funding acquisition, writing – review and editing. **Mohamed H. Al‐Thani:** data curation, resources, funding acquisition, writing – review and editing. **Abdullatif Al‐Khal:** data curation, resources, writing – review and editing, funding acquisition. **Roberto Bertollini:** data curation, resources, writing – review and editing, funding acquisition. **Laith J. Abu‐Raddad:** conceptualization, data curation, funding acquisition, investigation, methodology, project administration, resources, supervision, validation, writing – original draft, writing – review and editing.

## Ethics Statement

Hamad Medical Corporation and Weill Cornell Medicine–Qatar Institutional Review Boards approved this retrospective study with a waiver of informed consent.

## Conflicts of Interest

Adeel A. Butt has received institutional grant funding from Gilead Sciences unrelated to the work presented in this paper. The other authors declare no conflicts of interest.

### Peer Review

The peer review history for this article is available at https://www.webofscience.com/api/gateway/wos/peer‐review/10.1111/irv.13357.

## Supporting information


**Appendix S1.** Study population and data sources.
**Appendix S2.** Laboratory methods and variant ascertainment.
**Appendix S3.** COVID‐19 severity, criticality, and fatality classification.
**Appendix S4.** Classification of coexisting conditions.
**Appendix S5.** Matching of cohorts.
**Table S1.** Strengthening the Reporting of Observational Studies in Epidemiology (STROBE) checklist for cohort studies.
**Figure S1.** Flowchart describing the study population selection process for investigating the immune protection elicited by BNT162b2 versus mRNA‐1273 against SARS‐CoV‐2 infection and against severe forms of COVID‐19 after two or three vaccine doses.
**Figure S2.** Daily count of newly diagnosed SARS‐CoV‐2 infections between February 28, 2020 and the end of the study on February 18, 2024.
**Table S2.** Sensitivity analyses. Adjusted hazard ratios for incidence of SARS‐CoV‐2 infection additionally adjusted for differences in testing rate between the study cohorts or estimated using interaction terms between study cohort and prior infection status in the A) two‐dose analysis and B) three‐dose analysis.

## Data Availability

The dataset of this study is a property of the Qatar Ministry of Public Health that was provided to the researchers through a restricted access agreement that prevents sharing the dataset with a third party or publicly. The data are available under restricted access for preservation of confidentiality of patient data. Access can be obtained through a direct application for data access to Her Excellency the Minister of Public Health (https://www.moph.gov.qa/english/OurServices/eservices/Pages/Governmental‐HealthCommunication‐Center.aspx). The raw data are protected and are not available due to data privacy laws. Aggregate data are available within the paper and its supplementary information.
