# Supplementary material for: BNT162b2 Versus mRNA‐1273 Vaccines: Comparative Analysis of Long‐Term Protection Against SARS‐CoV‐2 Infection and Severe COVID‐19 in Qatar
Source: Influenza Other Respir Viruses. 2024 Sep 29;18(10):e13357. doi: 10.1111/irv.13357 (PMC11439586; doi:10.1111/irv.13357)
Supplement: Supplementary file 1 — Appendix S1. Study population and data sources. Appendix S2. Laboratory methods and variant ascertainment. Appendix S3. COVID‐19 severity, criticality, and fatality classification. Appendix S4. Classification of coexisting conditions. Appendix S5. Matching of cohorts. Table S1. Strengthening the Reporting of Observational Studies in Epidemiology (STROBE) checklist for cohort studies. Figure S1. Flowchart describing the study population selection process for investigating the immune protection elicited by BNT162b2 versus mRNA‐1273 against SARS‐CoV‐2 infection and against severe forms of COVID‐19 after two or three vaccine doses. Figure S2. Daily count of newly diagnosed SARS‐CoV‐2 infections between February 28, 2020 and the end of the study on February 18, 2024. Table S2. Sensitivity analyses. Adjusted hazard ratios for incidence of SARS‐CoV‐2 infection additionally adjusted for differences in testing rate between the study cohorts or estimated using interaction terms between study cohort and prior infection status in the A) two‐dose analysis and B) three‐dose analysis. [file IRV-18-e13357-s001.docx]

**Supplementary Appendix**

**Table of Contents**

[**SECTION S1. Study population and data sources** 2](#_Toc164281501)

[**SECTION S2. Laboratory methods and variant ascertainment.** 5](#_Toc164281502)

[**Real-time reverse-transcription polymerase chain reaction testing** 5](#_Toc164281503)

[**Rapid antigen testing** 5](#_Toc164281504)

[**Classification of infections by variant type** 6](#_Toc164281505)

[**SECTION S3. COVID-19 severity, criticality, and fatality classification.** 7](#_Toc164281506)

[**Severe COVID-19** 7](#_Toc164281507)

[**Critical COVID-19** 8](#_Toc164281508)

[**Fatal COVID-19** 8](#_Toc164281509)

[**SECTION S4. Classification of coexisting conditions** 9](#_Toc164281510)

[**SECTION S5. Matching of cohorts** 10](#_Toc164281511)

[**TABLE S1.** Strengthening the Reporting of Observational Studies in Epidemiology (STROBE) checklist for cohort studies. 11](#_Toc164281512)

[**FIGURE S1.** Flowchart describing the study population selection process for investigating the immune protection elicited by BNT162b2 versus mRNA-1273 against SARS-CoV-2 infection and against severe forms of COVID-19 after two or three vaccine doses. 13](#_Toc164281513)

[**FIGURE S2.** Daily count of newly diagnosed SARS-CoV-2 infections between February 28, 2020 and the end of the study on February 18, 2024. 14](#_Toc164281514)

[**TABLE S2.** Sensitivity analyses. Adjusted hazard ratios for incidence of SARS-CoV-2 infection additionally adjusted for differences in testing rate between the study cohorts or estimated using interaction terms between study cohort and prior infection status in the A) two-dose analysis and B) three-dose analysis. 15](#_Toc164281515)

[**REFERENCES** 16](#_Toc164281516)

# **SECTION S1.** **Study population and data sources**

Qatar's national and universal public healthcare system uses the Cerner-system advanced digital health platform to track all electronic health record encounters of each individual in the country, including all citizens and residents registered in the national and universal public healthcare system. Registration in the public healthcare system is mandatory for citizens and residents.

The databases analyzed in this study are data-extract downloads from the Cerner-system that have been implemented on a regular weekly schedule since the onset of pandemic by the Business Intelligence Unit at Hamad Medical Corporation (HMC). HMC is the national public healthcare provider in Qatar. At every download all severe acute respiratory syndrome coronavirus 2 (SARS-CoV-2) tests, coronavirus disease 2019 (COVID-19) vaccinations, hospitalizations related to COVID-19, and all death records regardless of cause are provided to the authors through .csv files. These databases have been analyzed throughout the pandemic not only for study-related purposes, but also to provide policymakers with summary data and analytics to inform the national response.

Every health encounter in the Cerner-system is linked to an individual through the HMC Number, which serves as a unique identifier that links all records for this individual at the national level. Databases were merged and analyzed using the HMC Number to link all records pertaining to testing, vaccinations, hospitalizations, and deaths. All deaths in Qatar are recorded by the public healthcare system. All COVID-19-related healthcare was provided exclusively in the public healthcare system. No private entity was permitted to provide COVID-19-related hospitalization. COVID-19 vaccination was also provided only through the public healthcare system. These health records were tracked throughout the COVID-19 pandemic using the Cerner system. This system has been implemented in 2013, before the onset of the pandemic. This pre-established system ensured that we had access to comprehensive health records related to this study for both citizens and residents throughout the entire pandemic, allowing us to follow each person over time.

Demographic details for every HMC Number (individual) such as sex, age, and nationality are collected upon issuing of the universal health card, based on the Qatar Identity Card, which is a mandatory requirement by the Ministry of Interior to every citizen and resident in the country. Data extraction from the Qatar Identity Card to the digital health platform is performed electronically through scanning techniques.

All SARS-CoV-2 testing in any facility in Qatar is tracked nationally in one database, the national testing database. This database covers all testing throughout the country, whether in public or private facilities. Every polymerase chain reaction (PCR) test and a proportion of the facility-based rapid antigen tests conducted in Qatar, regardless of location or setting, are classified on the basis of symptoms and the reason for testing, such as the presence of clinical symptoms, contact tracing, participation in surveys or random testing campaigns, individual requests for testing, routine healthcare testing, pre-travel requirements, at the point of entry into the country, or any other relevant reasons for testing.

Before November 1, 2022, SARS-CoV-2 testing in Qatar was performed extensively with about 5% of the population were tested every week.^1^ Based on the distribution of the reason for testing up to November 1, 2022, most of the tests in Qatar were conducted for routine reasons, such as travel-related purposes, and about 75% of infections were diagnosed not because of presence of symptoms.^1,2^ Starting from November 1, 2022, testing for SARS-CoV-2 was substantially reduced, but still close to 1% of the population are being tested every week.^2^ This study factored all SARS-CoV-2-related testing included in the national testing database over the duration of follow-up.

The first omicron wave that reached its peak in January of 2022 was massive and strained the testing capacity in the country.^1,3-5^ To alleviate the burden on PCR testing, rapid antigen testing was rapidly introduced. The swift change in testing policy precluded incorporating reason for testing for a number of rapid antigen tests. While the reason for testing is documented for all PCR tests, it is not uniformly available for all rapid antigen tests.

Rapid antigen test kits are accessible for purchase at pharmacies in Qatar, but results of home-based testing are neither reported nor documented in the national databases. Since SARS-CoV-2-test outcomes were linked to specific public health measures, restrictions, and privileges, testing policy and guidelines stress facility-based testing as the core testing mechanism in the population. While facility-based testing is provided free of charge or at low subsidized costs, depending on the reason for testing, home-based rapid antigen testing is de-emphasized and not supported as part of national policy.

Qatar launched its COVID-19 vaccination program in December 2020, employing mRNA vaccines and prioritizing individuals based on coexisting conditions and age criteria.^2,6^ COVID-19 vaccination was provided free of charge, regardless of citizenship or residency status, and was nationally tracked.^2,6^

Qatar has unusually young, diverse demographics, in that only 9% of its residents are ≥50 years of age, and 89% are expatriates from over 150 countries.^7,8^ Further descriptions of the study population and these national databases were reported previously.^1,2,5,8-12^

# **SECTION S2. Laboratory methods and variant ascertainment.**

## **Real-time reverse-transcription polymerase chain reaction testing**

Nasopharyngeal and/or oropharyngeal swabs were collected for PCR testing and placed in Universal Transport Medium (UTM). Aliquots of UTM were: 1) extracted on KingFisher Flex (Thermo Fisher Scientific, USA), MGISP-960 (MGI, China), or ExiPrep 96 Lite (Bioneer, South Korea) followed by testing with real-time reverse-transcription PCR (RT-qPCR) using TaqPath COVID-19 Combo Kits (Thermo Fisher Scientific, USA) on an ABI 7500 FAST (Thermo Fisher Scientific, USA); 2) tested directly on the Cepheid GeneXpert system using the Xpert Xpress SARS-CoV-2 (Cepheid, USA); or 3) loaded directly into a Roche cobas 6800 system and assayed with the cobas SARS-CoV-2 Test (Roche, Switzerland). The first assay targets the viral S, N, and ORF1ab gene regions. The second targets the viral N and E-gene regions, and the third targets the ORF1ab and E-gene regions.

All PCR testing was conducted at the Hamad Medical Corporation Central Laboratory or Sidra Medicine Laboratory, following standardized protocols.

## **Rapid antigen testing**

SARS-CoV-2 antigen tests were performed on nasopharyngeal swabs using one of the following lateral flow antigen tests: Panbio COVID-19 Ag Rapid Test Device (Abbott, USA); SARS-CoV-2 Rapid Antigen Test (Roche, Switzerland); Standard Q COVID-19 Antigen Test (SD Biosensor, Korea); or CareStart COVID-19 Antigen Test (Access Bio, USA). All antigen tests were performed point-of-care according to each manufacturer's instructions at public or private hospitals and clinics throughout Qatar with prior authorization and training by the Ministry of Public Health (MOPH). Antigen test results were electronically reported to the MOPH in real time using the Antigen Test Management System which is integrated with the national Coronavirus Disease 2019 (COVID-19) database.

## **Classification of infections by variant type**

Surveillance for SARS-CoV-2 variants in Qatar is based on viral genome sequencing and multiplex RT-qPCR variant screening^13^ of weekly collected random positive clinical samples,^2,14-18^ complemented by deep sequencing of wastewater samples.^16,19,20^ Further details on the viral genome sequencing and multiplex RT-qPCR variant screening throughout the SARS-CoV-2 waves in Qatar can be found in previous publications.^1,2,4,10,14-18,21-26^

# **SECTION S3. COVID-19 severity, criticality, and fatality classification.**

Classification of COVID-19 case severity (acute-care hospitalizations),^27^ criticality (intensive-care-unit hospitalizations),^27^ and fatality^28^ followed World Health Organization (WHO) guidelines. Assessments were made by trained medical personnel independent of study investigators and using individual chart reviews, as part of a national protocol applied to every hospitalized COVID-19 patient. Each hospitalized COVID-19 patient underwent an infection severity assessment every three days until discharge or death. We classified individuals who progressed to severe, critical, or fatal COVID-19 between the time of the documented infection and the end of the study based on their worst outcome, starting with death,^28^ followed by critical disease,^27^ and then severe disease.^27^

## **Severe COVID-19**

Severe COVID-19 disease was defined per WHO classification as a SARS-CoV-2 infected person with “oxygen saturation of <90% on room air, and/or respiratory rate of >30 breaths/minute in adults and children >5 years old (or ≥60 breaths/minute in children <2 months old or ≥50 breaths/minute in children 2-11 months old or ≥40 breaths/minute in children 1–5 years old), and/or signs of severe respiratory distress (accessory muscle use and inability to complete full sentences, and, in children, very severe chest wall indrawing, grunting, central cyanosis, or presence of any other general danger signs)”.^27^ Detailed WHO criteria for classifying Severe acute respiratory syndrome coronavirus 2 (SARS-CoV-2) infection severity can be found in the WHO technical report.^27^

## **Critical COVID-19**

Critical COVID-19 disease was defined per WHO classification as a SARS-CoV-2 infected person with “acute respiratory distress syndrome, sepsis, septic shock, or other conditions that would normally require the provision of life sustaining therapies such as mechanical ventilation (invasive or non-invasive) or vasopressor therapy”.^27^ Detailed WHO criteria for classifying SARS-CoV-2 infection criticality can be found in the WHO technical report.^27^

## **Fatal COVID-19**

COVID-19 death was defined per WHO classification as “a death resulting from a clinically compatible illness, in a probable or confirmed COVID-19 case, unless there is a clear alternative cause of death that cannot be related to COVID-19 disease (e.g. trauma). There should be no period of complete recovery from COVID-19 between illness and death. A death due to COVID-19 may not be attributed to another disease (e.g. cancer) and should be counted independently of preexisting conditions that are suspected of triggering a severe course of COVID-19”. Detailed WHO criteria for classifying COVID-19 death can be found in the WHO technical report.^28^

# **SECTION S4. Classification of coexisting conditions**

Coexisting conditions were ascertained and classified based on the ICD-10 codes for the conditions as recorded in the electronic health record encounters of each individual in the Cerner-system national database that includes all citizens and residents registered in the national and universal public healthcare system. The public healthcare system provides healthcare to the entire resident population of Qatar free of charge or at heavily subsidized costs, including prescription drugs. With the mass expansion of this sector in recent years, facilities have been built to cater to specific needs of subpopulations. For example, tens of facilities have been built, including clinics and hospitals, in localities with high density of craft and manual workers.^29^

All encounters for each individual were analyzed to determine the coexisting-condition classification for that individual. The Cerner-system national database includes encounters starting from 2013, after this system was launched in Qatar. As long as each individual had at least one encounter with a specific coexisting-condition diagnosis since 2013, this person was classified with this coexisting condition.

Individuals who have coexisting conditions but never sought care in the public healthcare system, or seek care exclusively in private healthcare facilities, were classified as individuals with no coexisting condition due to absence of recorded encounters for them.

# **SECTION S5. Matching of cohorts**

Each individual in the BNT162b2 cohort was matched to an individual in the mRNA-1273 cohort exactly one-to-one by sex, 10-year age group, nationality, number of coexisting conditions, prior infection status, and calendar week of the second vaccine dose for the two-dose analysis and of the third dose for the three-dose analysis.

Matching was implemented using an iterative algorithm that selected individuals from the reference cohort (vaccinated with mRNA-1273) ensuring that, at the start of the follow-up, they were alive, had the same vaccination status (primary-series or three doses) and prior infection status as their match, and no documented SARS-CoV-2 infection within the previous 90 days. The matching algorithm was implemented using *ccmatch* command in Stata supplemented with conditions to retain only controls that fulfilled these eligibility criteria and was iterated using loops with as many replications as needed until exhaustion (i.e., no more matched pairs could be identified).

Individuals in the matched primary-series cohorts contributed follow-up time before receiving the third dose/booster vaccination, that is, in the two-dose analysis, and subsequently contributed follow-up time in the three-dose analysis if they received a third dose.

# **TABLE S1.** Strengthening the Reporting of Observational Studies in Epidemiology (STROBE) checklist for cohort studies.

|  | Item No | Recommendation | Main Text page |
| --- | --- | --- | --- |
| **Title and abstract** | 1 | (*a*) Indicate the study’s design with a commonly used term in the title or the abstract | Abstract |
|  |  | (*b*) Provide in the abstract an informative and balanced summary of what was done and what was found | Abstract |
| Introduction | | | |
| Background/rationale | 2 | Explain the scientific background and rationale for the investigation being reported | Introduction |
| Objectives | 3 | State specific objectives, including any prespecified hypotheses | Introduction |
| Methods | | | |
| Study design | 4 | Present key elements of study design early in the paper | Methods (‘Study population, data sources, and vaccination’ & ‘Study design’) |
| Setting | 5 | Describe the setting, locations, and relevant dates, including periods of recruitment, exposure, follow-up, and data collection | Methods (‘Study population, data sources, and vaccination’ & ‘Study design’) & Figure S1 in Supplementary Appendix |
| Participants | 6 | (*a*) Give the eligibility criteria, and the sources and methods of selection of participants. Describe methods of follow-up | Methods (‘Cohorts’ eligibility and matching’), & Figure S1 & Section S5 in Supplementary Appendix |
|  |  | (*b*) For matched studies, give matching criteria and number of exposed and unexposed |  |
| Variables | 7 | Clearly define all outcomes, exposures, predictors, potential confounders, and effect modifiers. Give diagnostic criteria, if applicable | Methods (‘Study design’, ‘Cohorts’ eligibility and matching’, ‘Cohorts’ follow-up’, & ‘Statistical analysis’), Table 1, & Sections S1-S5 in Supplementary Appendix |
| Data sources/ measurement | 8* | For each variable of interest, give sources of data and details of methods of assessment (measurement). Describe comparability of assessment methods if there is more than one group | Methods (‘Study population, data sources, and vaccination’, ‘Study design’, & ‘Statistical analysis’), Table 1, & Sections S1-S4 in Supplementary Appendix |
| Bias | 9 | Describe any efforts to address potential sources of bias | Methods (‘Cohorts’ eligibility and matching’, ‘Cohorts’ follow-up’, & ‘Statistical analysis’) & Section S5 in Supplementary Appendix |
| Study size | 10 | Explain how the study size was arrived at | Figure S1 in Supplementary Appendix |
| Quantitative variables | 11 | Explain how quantitative variables were handled in the analyses. If applicable, describe which groupings were chosen and why | Methods (‘Cohorts’ eligibility and matching’ & ‘Statistical analysis’) & Table 1 |
| Statistical methods | 12 | (*a*) Describe all statistical methods, including those used to control for confounding | Methods (‘Statistical analysis’) |
|  |  | (*b*) Describe any methods used to examine subgroups and interactions | Methods (‘Statistical analysis’) |
|  |  | (*c*) Explain how missing data were addressed | Not applicable, see Methods (‘Study population, data sources, and vaccination’) & Section S1 in Supplementary Appendix |
|  |  | (*d*) If applicable, explain how loss to follow-up was addressed | Not applicable, see Methods (‘Study population, data sources, and vaccination’) & Section S1 in Supplementary Appendix |
|  |  | (*e*) Describe any sensitivity analyses | Methods (‘Statistical analysis’) |
| Results | | |  |
| Participants | 13* | (a) Report numbers of individuals at each stage of study—eg numbers potentially eligible, examined for eligibility, confirmed eligible, included in the study, completing follow-up, and analysed | Results (‘Study population’), Table 1, & Figure S1 in Supplementary Appendix |
|  |  | (b) Give reasons for non-participation at each stage |  |
|  |  | (c) Consider use of a flow diagram |  |
| Descriptive data | 14 | (a) Give characteristics of study participants (eg demographic, clinical, social) and information on exposures and potential confounders | Results (‘Study population’), Table 1, & Figure S1 in Supplementary Appendix |
|  |  | (b) Indicate number of participants with missing data for each variable of interest | Not applicable, see Methods (‘Study population, data sources, and vaccination’) & Section S1 in Supplementary Appendix |
|  |  | (c) Summarise follow-up time (eg, average and total amount) | Results (‘Two-dose analysis’, paragraph 1 & ‘Three-dose analysis, paragraph 1’), Figure 1, & Table 2 |
| Outcome data | 15 | Report numbers of outcome events or summary measures over time | Results (‘Two-dose analysis’, paragraphs 1-2 & ‘Three-dose analysis, paragraphs 1-2’), Table 2, & Figure S1 in Supplementary Appendix |
| Main results | 16 | (a) Give unadjusted estimates and, if applicable, confounder-adjusted estimates and their precision (eg, 95% confidence interval). Make clear which confounders were adjusted for and why they were included | Results (‘Two-dose analysis’, paragraph 3 & ‘Three-dose analysis, paragraph 2’) & Table 2 |
|  |  | (b) Report category boundaries when continuous variables were categorized | Table 1 |
|  |  | (c) If relevant, consider translating estimates of relative risk into absolute risk for a meaningful time period | Not applicable |
| Other analyses | 17 | Report other analyses done—eg analyses of subgroups and interactions, and sensitivity analyses | Results (‘Two-dose analysis’, paragraphs 4-6 & ‘Three-dose analysis, paragraphs 4-6’), Figure 2,, & Table S2 in Supplementary Appendix |
| Discussion | | | |
| Key results | 18 | Summarise key results with reference to study objectives | Discussion, paragraphs 1-5 |
| Limitations | 19 | Discuss limitations of the study, taking into account sources of potential bias or imprecision. Discuss both direction and magnitude of any potential bias | Discussion, paragraphs 6-11 |
| Interpretation | 20 | Give a cautious overall interpretation of results considering objectives, limitations, multiplicity of analyses, results from similar studies, and other relevant evidence | Discussion, paragraph 13 |
| Generalisability | 21 | Discuss the generalisability (external validity) of the study results | Discussion, paragraph 8 |
| Other information | | | |
| Funding | 22 | Give the source of funding and the role of the funders for the present study and, if applicable, for the original study on which the present article is based | Acknowledgements |

# **FIGURE S1.** Flowchart describing the study population selection process for investigating the immune protection elicited by BNT162b2 versus mRNA-1273 against SARS-CoV-2 infection and against severe forms of COVID-19 after two or three vaccine doses.


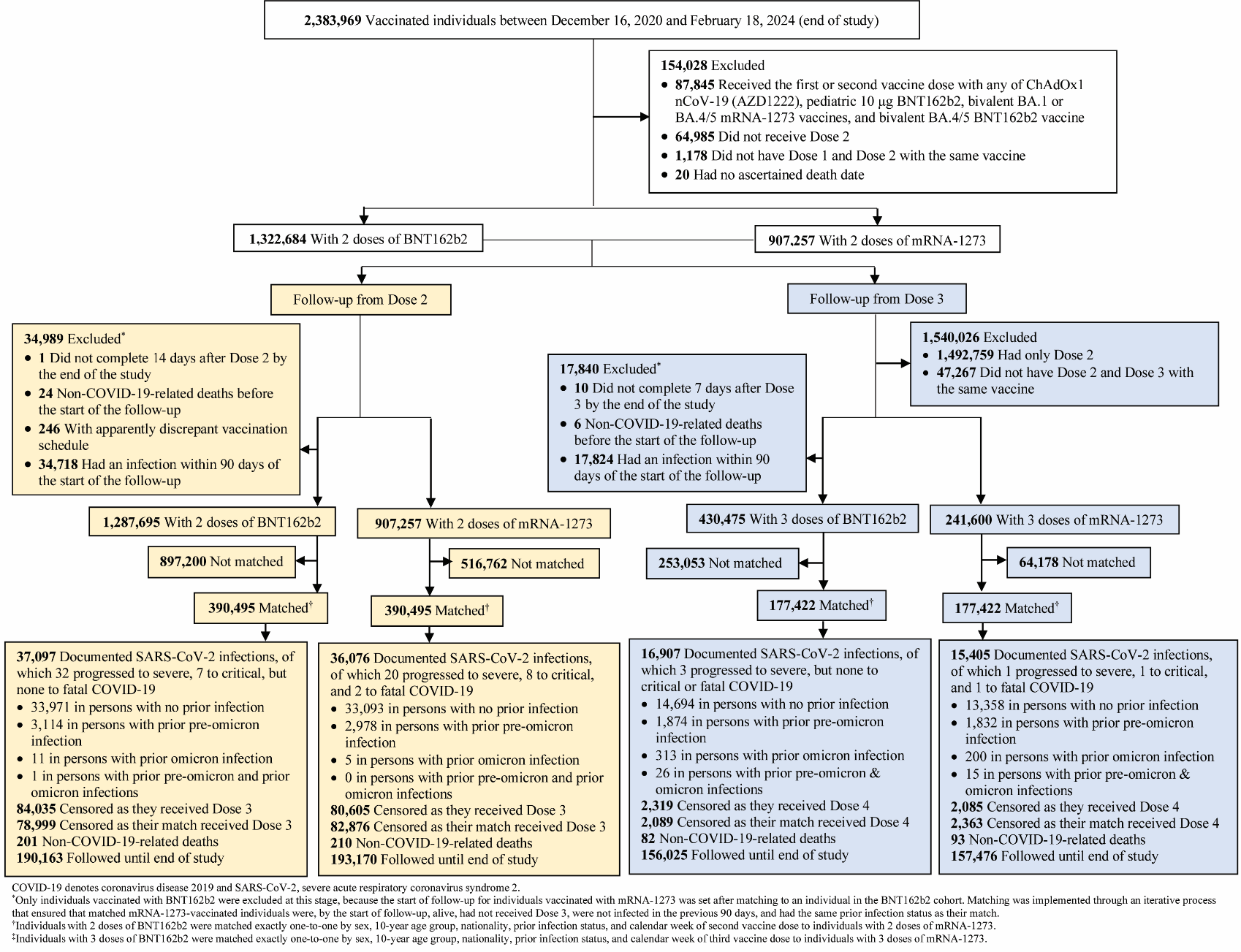


# **FIGURE S2.** Daily count of newly diagnosed SARS-CoV-2 infections between February 28, 2020 and the end of the study on February 18, 2024.


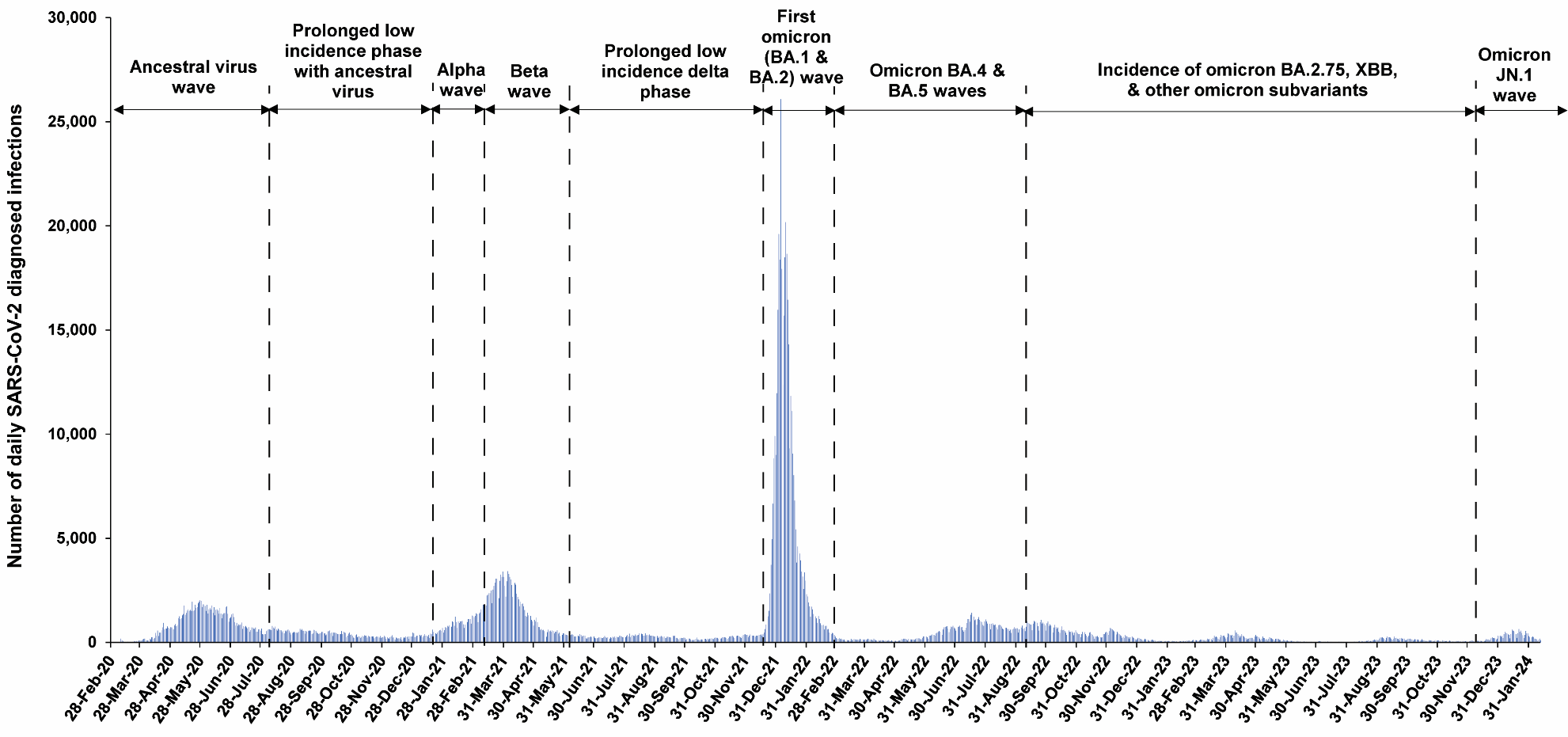


# **TABLE S2.** Sensitivity analyses. Adjusted hazard ratios for incidence of SARS-CoV-2 infection additionally adjusted for differences in testing rate between the study cohorts or estimated using interaction terms between study cohort and prior infection status in the A) two-dose analysis and B) three-dose analysis.

| 1. **Two-dose analysis** | **BNT162b2 cohort^*^** | **mRNA-1273 cohort^*^** |
| --- | --- | --- |
| **Main analysis** |  |  |
| Adjusted hazard ratio for SARS-CoV-2 infection (95% CI)^†^ | 1.03 (1.01 to 1.04) | |
| **Subgroup analyses** | | |
| **No prior infection** | | |
| Adjusted hazard ratio for SARS-CoV-2 infection (95% CI)^†^ | 1.03 (1.01 to 1.04) | |
| Adjusted hazard ratio for SARS-CoV-2 infection using interaction (95% CI)^‡^ | 1.03 (1.02 to 1.05) | |
| **Prior pre-omicron infection** | | |
| Adjusted hazard ratio for SARS-CoV-2 infection (95% CI)^†^ | 1.03 (0.98 to 1.08) | |
| Adjusted hazard ratio for SARS-CoV-2 infection using interaction (95% CI)^‡^ | 1.05 (1.00 to 1.10) | |
| **Prior omicron infection** | | |
| Adjusted hazard ratio for SARS-CoV-2 infection (95% CI)^†^ | 2.43 (0.84 to 7.02) | |
| Adjusted hazard ratio for SARS-CoV-2 infection using interaction (95% CI)^‡^ | 2.26 (0.78 to 6.49) | |
| **Prior pre-omicron & omicron infections** | | |
| Adjusted hazard ratio for SARS-CoV-2 infection (95% CI)^†^ | -- | |
| Adjusted hazard ratio for SARS-CoV-2 infection using interaction (95% CI)^‡^ | -- | |
| 1. **Three-dose analysis** | **BNT162b2 cohort^*^** | **mRNA-1273 cohort^*^** |
| **Main analysis** |  |  |
| Adjusted hazard ratio for SARS-CoV-2 infection (95% CI)^†^ | 1.05 (1.03 to 1.07) | |
| **Subgroup analyses** | | |
| **No prior infection** | | |
| Adjusted hazard ratio for SARS-CoV-2 infection (95% CI)^†^ | 1.05 (1.03 to 1.08) | |
| Adjusted hazard ratio for SARS-CoV-2 infection using interaction (95% CI)^‡^ | 1.11 (1.09 to 1.14) | |
| **Prior pre-omicron infection** | | |
| Adjusted hazard ratio for SARS-CoV-2 infection (95% CI)^†^ | 0.98 (0.93 to 1.05) | |
| Adjusted hazard ratio for SARS-CoV-2 infection using interaction (95% CI)^‡^ | 1.02 (0.96 to 1.09) | |
| **Prior omicron infection** | | |
| Adjusted hazard ratio for SARS-CoV-2 infection (95% CI)^†^ | 1.40 (1.17 to 1.68) | |
| Adjusted hazard ratio for SARS-CoV-2 infection using interaction (95% CI)^‡^ | 1.58 (1.32 to 1.88) | |
| **Prior pre-omicron & omicron infections** | | |
| Adjusted hazard ratio for SARS-CoV-2 infection (95% CI)^†^ | 1.92 (1.00 to 3.69) | |
| Adjusted hazard ratio for SARS-CoV-2 infection using interaction (95% CI)^‡^ | 1.77 (0.94 to 3.35) | |

CI denotes confidence interval, and SARS-CoV-2, severe acute respiratory syndrome coronavirus 2.

^*^Cohorts were matched exactly one-to-one by sex, 10-year age group, nationality, number of coexisting conditions, prior infection status, and calendar week of the second vaccine dose in the two-dose analysis and calendar week of the third dose in the three-dose analysis.

^†^Adjusted for sex, 10-year age group, nationality, number of coexisting conditions, and calendar week of the second vaccine dose in the two-dose analysis and calendar week of the third dose in the three-dose analysis, as well as differences in testing rate between the study cohorts.

^‡^Adjusted for sex, 10-year age group, nationality, number of coexisting conditions, and calendar week of the second vaccine dose in the two-dose analysis and calendar week of the third dose in the three-dose analysis.

# **REFERENCES**

1. Altarawneh HN, Chemaitelly H, Ayoub HH, et al. Effects of Previous Infection and Vaccination on Symptomatic Omicron Infections. *N Engl J Med.* 2022;387(1):21-34.

2. Chemaitelly H, Tang P, Hasan MR, et al. Waning of BNT162b2 Vaccine Protection against SARS-CoV-2 Infection in Qatar. *N Engl J Med.* 2021;385(24):e83.

3. Chemaitelly H, Ayoub HH, AlMukdad S, et al. Bivalent mRNA-1273.214 vaccine effectiveness against SARS-CoV-2 omicron XBB* infections. *J Travel Med.* 2023;30(5).

4. Altarawneh HN, Chemaitelly H, Hasan MR, et al. Protection against the Omicron Variant from Previous SARS-CoV-2 Infection. *N Engl J Med.* 2022;386(13):1288-1290.

5. Chemaitelly H, Ayoub HH, Tang P, et al. Long-term COVID-19 booster effectiveness by infection history and clinical vulnerability and immune imprinting: a retrospective population-based cohort study. *Lancet Infect Dis.* 2023;23(7):816-827.

6. Abu-Raddad LJ, Chemaitelly H, Bertollini R, National Study Group for Covid Vaccination. Effectiveness of mRNA-1273 and BNT162b2 Vaccines in Qatar. *N Engl J Med.* 2022;386(8):799-800.

7. Planning and Statistics Authority-State of Qatar. Qatar Monthly Statistics. Available from: <https://www.psa.gov.qa/en/pages/default.aspx>. Accessed on: May 26, 2020. 2020.

8. Abu-Raddad LJ, Chemaitelly H, Ayoub HH, et al. Characterizing the Qatar advanced-phase SARS-CoV-2 epidemic. *Sci Rep.* 2021;11(1):6233.

9. Chemaitelly H, Bertollini R, Abu-Raddad LJ, National Study Group for Covid Epidemiology. Efficacy of Natural Immunity against SARS-CoV-2 Reinfection with the Beta Variant. *N Engl J Med.* 2021;385(27):2585-2586.

10. Abu-Raddad LJ, Chemaitelly H, Ayoub HH, et al. Effect of mRNA Vaccine Boosters against SARS-CoV-2 Omicron Infection in Qatar. *N Engl J Med.* 2022;386(19):1804-1816.

11. Chemaitelly H, Faust JS, Krumholz HM, et al. Short- and longer-term all-cause mortality among SARS-CoV-2- infected individuals and the pull-forward phenomenon in Qatar: a national cohort study. *Int J Infect Dis.* 2023;136:81-90.

12. AlNuaimi AA, Chemaitelly H, Semaan S, et al. All-cause and COVID-19 mortality in Qatar during the COVID-19 pandemic. *BMJ Glob Health.* 2023;8(5).

13. Vogels C, Fauver J, Grubaugh N. Multiplexed RT-qPCR to screen for SARS-COV-2 B.1.1.7, B.1.351, and P.1 variants of concern V.3. dx.doi.org/10.17504/protocols.io.br9vm966. 2021(June 6, 2021).

14. Abu-Raddad LJ, Chemaitelly H, Butt AA, National Study Group for Covid Vaccination. Effectiveness of the BNT162b2 Covid-19 Vaccine against the B.1.1.7 and B.1.351 Variants. *N Engl J Med.* 2021;385(2):187-189.

15. Chemaitelly H, Yassine HM, Benslimane FM, et al. mRNA-1273 COVID-19 vaccine effectiveness against the B.1.1.7 and B.1.351 variants and severe COVID-19 disease in Qatar. *Nat Med.* 2021;27(9):1614-1621.

16. National Project of Surveillance for Variants of Concern and Viral Genome Sequencing. Qatar viral genome sequencing data. Data on randomly collected samples. <https://www.gisaid.org/phylodynamics/global/nextstrain/>. 2021; <https://www.gisaid.org/phylodynamics/global/nextstrain/>.

17. Benslimane FM, Al Khatib HA, Al-Jamal O, et al. One Year of SARS-CoV-2: Genomic Characterization of COVID-19 Outbreak in Qatar. *Front Cell Infect Microbiol.* 2021;11:768883.

18. Hasan MR, Kalikiri MKR, Mirza F, et al. Real-Time SARS-CoV-2 Genotyping by High-Throughput Multiplex PCR Reveals the Epidemiology of the Variants of Concern in Qatar. *Int J Infect Dis.* 2021;112:52-54.

19. Saththasivam J, El-Malah SS, Gomez TA, et al. COVID-19 (SARS-CoV-2) outbreak monitoring using wastewater-based epidemiology in Qatar. *Sci Total Environ.* 2021;774:145608.

20. El-Malah SS, Saththasivam J, Jabbar KA, et al. Application of human RNase P normalization for the realistic estimation of SARS-CoV-2 viral load in wastewater: A perspective from Qatar wastewater surveillance. *Environ Technol Innov.* 2022;27:102775.

21. Tang P, Hasan MR, Chemaitelly H, et al. BNT162b2 and mRNA-1273 COVID-19 vaccine effectiveness against the SARS-CoV-2 Delta variant in Qatar. *Nat Med.* 2021;27(12):2136-2143.

22. Chemaitelly H, Ayoub HH, AlMukdad S, et al. Duration of mRNA vaccine protection against SARS-CoV-2 Omicron BA.1 and BA.2 subvariants in Qatar. *Nat Commun.* 2022;13(1):3082.

23. Qassim SH, Chemaitelly H, Ayoub HH, et al. Effects of BA.1/BA.2 subvariant, vaccination and prior infection on infectiousness of SARS-CoV-2 omicron infections. *J Travel Med.* 2022;29(6).

24. Altarawneh HN, Chemaitelly H, Ayoub HH, et al. Protective Effect of Previous SARS-CoV-2 Infection against Omicron BA.4 and BA.5 Subvariants. *N Engl J Med.* 2022;387(17):1620-1622.

25. Chemaitelly H, Tang P, Coyle P, et al. Protection against Reinfection with the Omicron BA.2.75 Subvariant. *N Engl J Med.* 2023;388(7):665-667.

26. Chemaitelly H, Coyle P, Kacem MAB, et al. Protection of natural infection against reinfection with SARS-CoV-2 JN.1 variant. *medRxiv.* 2024:2024.2002.2022.24303193.

27. World Health Organization (WHO). Living guidance for clinical management of COVID-19. Aavailable from: <https://www.who.int/publications/i/item/WHO-2019-nCoV-clinical-2021-2>. Accessed on: February 27, 2023. 2021.

28. World Health Organization (WHO). International Guidelines for Certification and Classification (Coding) of COVID-19 as Cause of Death. Available from: <https://www.who.int/publications/m/item/international-guidelines-for-certification-and-classification-(coding)-of-covid-19-as-cause-of-death>. Accessed on: February 27, 2023. 2020.

29. Al-Thani MH, Farag E, Bertollini R, et al. SARS-CoV-2 Infection Is at Herd Immunity in the Majority Segment of the Population of Qatar. *Open Forum Infect Dis.* 2021;8(8):ofab221.
